# Evaluation of early mini-bronchoalveolar lavage in the diagnosis of health care-associated pneumonia: a prospective study

**DOI:** 10.1186/cc12501

**Published:** 2013-02-05

**Authors:** Guillaume Lacroix, Bertrand Prunet, Julien Bordes, Nathalie Cabon-Asencio, Yves Asencio, Tiphaine Gaillard, Sandrine Pons, Erwan D'aranda, Delphine Kerebel, Eric Meaudre, Philippe Goutorbe

**Affiliations:** 1 Department of Anesthesiology and Intensive Care, Sainte Anne Military Teaching Hospital, Boulevard Sainte Anne, BP 20545, 83041 Cedex 09 Toulon France; 2 Medical Records Department, Sainte Anne Military Teaching Hospital, Boulevard Sainte Anne, BP 20545, 83041 Cedex 09 Toulon France; 3 Bacteriological laboratory federation, Sainte Anne Military Teaching Hospital, Boulevard Sainte Anne, BP 20545, 83041 Cedex 09 Toulon France

## Abstract

**Introduction:**

Health care-associated pneumonia (HCAP) has been proposed as a new category of respiratory infection to identify patients at risk of multidrug-resistant (MDR) pathogens. The American Thoracic Society's recommendation for HCAP treatment is to use broad-spectrum and multiple antibiotics. However, this strategy may be economically expensive and promote antimicrobial resistance when a multisensitive pathogen is not identified.

**Methods:**

We prospectively included all patients presenting with HCAP in the emergency department. Blood cultures and fiberoptic bronchoscope-guided distal protected small volume bronchoalveolar lavage (FODP mini-BAL) were performed in each patient. Empirical antibiotic therapy was adapted when microbiological findings were available. The primary objective was to assess whether FODP mini-BAL is more efficient than blood cultures in identifying pathogens with the ratio of identification between both techniques as principal criteria.

**Results:**

We included 54 patients with HCAP. Pathogens were identified in 46.3% of cases using mini-BAL and in 11.1% of cases using blood cultures (*P *<0.01). When the patient did not receive antibiotic therapy before the procedure, pathogens were identified in 72.6% of cases using mini-BAL and in 9.5% of cases using blood cultures (*P *<0.01). We noted multidrug-resistant pathogens in 16% of cases. All bronchoscopic procedures could be performed in patients without complications.

**Conclusions:**

FODP mini-BAL was more efficient than blood cultures for identifying pathogens in patients presenting with HCAP. When bacteriological identification was obtained, antibiotic therapy was adapted in 100% of cases.

See related letter by Sircar * et al.,*http://ccforum.com/content/17/2/428

## Introduction

Health care-associated pneumonia (HCAP) is associated with higher mortality than community-acquired pneumonia, because patients presenting with HCAP are at risk of multidrug-resistant (MDR) pathogens and seem to receive initially inappropriate therapy [1]. Indeed, empirical antibiotic therapy recommended for the management of community-acquired pneumonia is not adapted [2,3]. That is why guidelines recommend the use of broad-spectrum and multiple antibiotics in patients presenting with HCAP [4].

However, some recent studies focus on the fact that HCAP does not correlate well with the presence of resistant pathogens and that can lead to unnecessary antibiotic prescription with economic and ecological consequences [5]. That is why accurate microbiological identification is essential in the management of HCAP to de-escalate antibiotic therapy [6]. Actually, bacteriological diagnosis in pneumonia is based on blood cultures. Sputum culture may be sensitive for the diagnosis of pathogens but it is no longer performed for the diagnosis of pneumonia (except tuberculosis) in our hospital because of its lack of specificity. However, the identification rate using blood cultures remains low, up to 3.4% in an emergency department [7]. Mini-bronchoalveolar lavage (BAL) has been shown to be a useful tool in identifying pathogens, as in ventilator-associated pneumonia, with identification in up to 46.2% of cases, or in acute hypoxemic respiratory failure requiring noninvasive ventilation [8,9].

We conducted a prospective study to assess whether fiberoptic bronchoscope-guided distal-protected small volume bronchoalveolar lavage (FODP mini-BAL) with quantitative cultures was more efficient than blood cultures to identify pathogens in patients presenting with HCAP with the ratio of identification between the techniques as principal criteria.

## Material and methods

### Study design

The study was a prospective cohort and was approved by the local hospital's ethics committee. Patients admitted between February 2008 and February 2010 to the emergency department of Sainte Anne Military Teaching Hospital, Toulon, France, were eligible for enrollment into the study. Informed consent was obtained from each patient.

### Patients

We included all patients in the emergency department who were diagnosed with HCAP. Pneumonia was defined as the presence of a new infiltrate on a chest radiograph plus one or more of the following: fever (temperature >38.0°C) or hypothermia (temperature <35.0°C); new cough with or without sputum production; pleuritic chest pain; dyspnea; and altered breath sounds on auscultation [6]. HCAP was defined by pneumonia and one or more of the four criteria defined by the American Thoracic Society (ATS) [2]: hospitalized for two or more days in an acute care facility within 90 days before infection; resident of a nursing home or long-term care facility; attending a hospital or hemodialysis clinic, or received recent intravenous antimicrobial therapy, immunosuppressive therapy, or wound care within 30 days of infection. The exclusion criteria were: age younger than 18 years; use of antiplatelet drugs; use of anti-vitamin K medications; coagulation failure; opposition from the patient; judiciary protection of the patient; and bronchospasm.

### Diagnostic procedure

The diagnostic procedure was performed after initial symptomatic treatment. Evaluation of level of care and respiratory support needed by the patient was made by the intensivist on clinical grounds and according to published guidelines. Microbiological diagnosis was made as soon as possible, using FODP mini-BAL and blood cultures for each patient performed at the same time. Positive pathogen identification with mini-BAL was defined as >10^3^CFU/mL [8,10].

**Figure 1:**
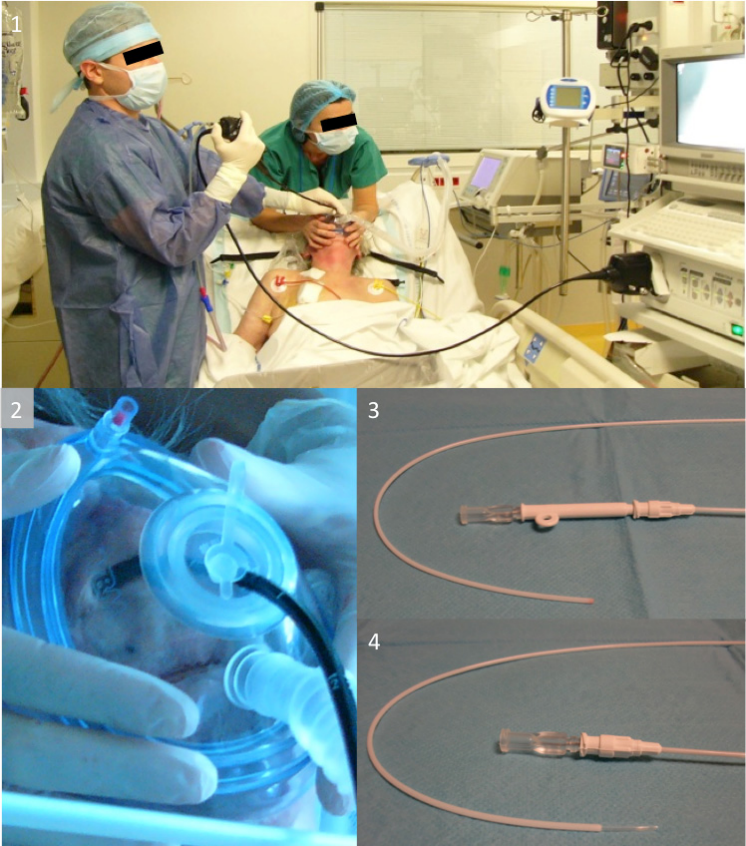
**Fiberoptic bronchoscope-guided distal protected small volume bronchoalveolar lavage (FODP mini-BAL)**. 1) FODP mini-BAL during non invasive ventilation. 2) Specific interface to perform fiberoptic bronchoscopy during non invasive ventilation. 3) Telescopic catheter Combicath^® ^before deployment. 4) Telescopic catheter Combicath^® ^deployed.

All patients were on non-invasive ventilation (NIV) (BiPAP Vision^®^, Philips Respitronics, Amsterdam, Netherlands) throughout the FODP mini-BAL, except for those who required endotracheal intubation prior to the procedure (Figure 1). The procedure was performed in the emergency department when the patient did not initially require tracheal intubation. The fiberoptic bronchoscope was positioned in the lobar bronchia where the pneumonia was localized on chest x-ray. The telescopic catheter (Combicath^®^, Prodimed division Plastimed, Le Plessis-Bouchard, FRANCE) was passed through the bronchoscope operator channel. The internal catheter was placed in the distal position and 20 mL of normal saline was infused, then secretions were removed with a syringe [10-12]. Two series of aerobic and anaerobic blood cultures were conducted at the same time. Blood samples were immediately taken to the laboratory.

### Antibiotic therapy

Broad-spectrum antibiotic combination with piperacillin-tazobactam, ciprofloxacin, and vancomycin was started as soon as possible according to ATS recommendations [4]. Treatment was adapted when microbiological results were available. When no pathogen was identified, initial treatment was prolonged to 14 days [2].

De-escalation is defined by adaptation from a broad-spectrum antibiotics combination therapy to a targeted and shortened treatment guided by antibiogram. When identification and antibiogram were available, the duration of treatment was adapted to the identified bacteria: 7 days in all cases except for *Pseudomonas aeruginosa *(14 days), *Legionnella pneumophilia *(21 days) and *Mycobacterium tuberculosis *(6 months).

### Data recorded

We recorded the patients' characteristics, including sex ratio, Fine score, and HCAP criteria (Table 1). We used the Fine score to categorize the severity of pneumonia [13,14].

**Table 1:** Patients' characteristics

	Total
Number	54
Sex ratio (male/female)	31/23
Age (years) Age >80 years	69.5 ± 6.5 14(26%)
Length of hospital stay (days)	15.9 ± 7.6
30 day survey Pneumonia related mortality Mortality (other causes)	39 (72.2%) 4(7.4%) 11(20.4%)
Fine mean ± SD	148 ± 19
Fine group	
I	0(0%)
II	0(0%)
III	2(4%)
IV	21(39%)
V	31(57%)
CURB 65 mean ± SD	1.6 ± 1
CURB 65 stratum:	
0	7(13%)
1	19(35%)
2	17(31%)
3	9(17%)
4	2(4%)
5	0(0%)
Ventilator support	
Non-invasive ventilation	37 (68%)
Tracheal intubation	17 (32%)
Comorbidities	
Neoplasic disease	19 (35.2%)
Liver disease	3 (5.6%)
Congestive heart failure	12 (22.2%)
Cerebrovascular disease	4 (7.4%)
Renal disease	13 (24.1%)
Physical examination findings	
Altered mental status	17 (31.5%)
Respiratory rate >30 breaths/min	31 (57.4%)
Systolic blood pressure <90 mmHg	11 (20.4%)
Pulse >125 beats/min	28 (51.9%)
Laboratory and radiographic findings	
Arterial pH <7.35	16 (29.6%)
Blood urea nitrogen ≥30 mg/dL	29 (53.7%)
Sodium <130 mmol/L	10 (18.5%)
Glucose ≥250 mg/dL (14 mmol/L)	6 (11.1%)
Hematocrit <30%	5 (9.3%)
Partial pressure of arterial oxygen <60 mmHg	35 (64.8%)
Pleural effusion	12 (22.2%)
HCAP Criteria:	
Resident of a nursing home or long-term care facility	6 (11.1%)
Hospitalized for two or more days in an acute care facility within 90 days of infection	14 (26%)
Attended a hospital or hemodialysis clinic or received recent intravenous antimicrobial therapy, immunosuppressive therapy, or wound care within 30 days of infection	33 (61%)
Nursing, intravenous infusion, or wound care at home within 30 days of infection	16 (29.6%)

Complications possibly related to bronchoscopy were categorized as follows: death in the first six hours; requiring tracheal intubation in the first six hours; requiring more than six hours of continuous NIV after mini-BAL without requiring NIV before mini-BAL; hemoptysis; and pneumothorax.

### Statistical analysis

The primary endpoint was to compare the pathogen identification rate of two microbiological techniques: blood cultures versus FODP mini-BAL. Deschamps *et al.* reported a 40% identification rate with BAL and 5.7% with blood cultures in patients with hospital-acquired pneumonia [15]. According to these previous results, we calculated that 49 patients were required in order to have an 80% power for detecting a 25% absolute difference in pathogen identification between the fiberoptic and blood culture techniques with a two-sided chi-square test and α set at 0.05. Continuous variables were compared using paired or unpaired Wilcoxon rank-sum tests. Categorical variables were compared using the chi-square test or Fisher's exact test when appropriate. A *P*-value < .05 was considered statistically significant. All statistical analyses were performed using JMP 8.0.1 statistical software (SAS Institute, Cary, NC, USA).

## Results

### Patient characteristics

**Figure 2:**
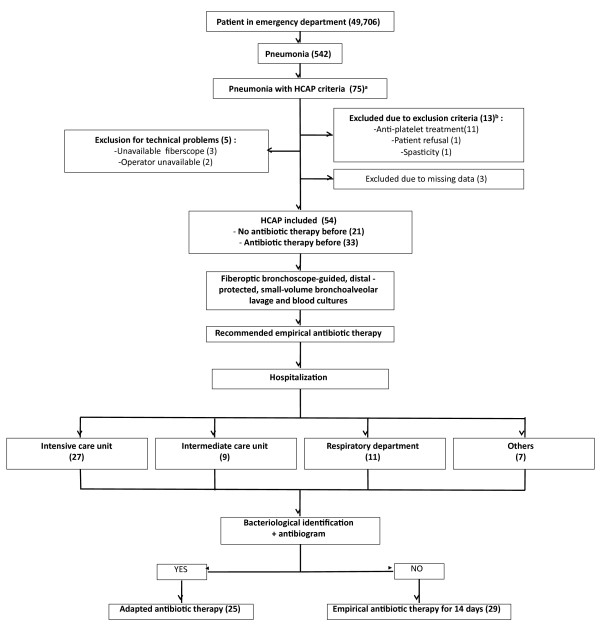
**Flow chart**. **(a) **HCAP criteria: Hospitalized for two or more days in an acute care facility within 90 days of infection; resident of a nursing home or long-term care facility; attended a hospital or hemodialysis clinic, or received recent intravenous antimicrobial therapy, immunosuppressive therapy, or wound care within 30 days of infection**. (b) **Exclusion criteria: Younger than 18 years; patient refusal; coagulation disorders; judiciary protected patient; bronschospasm.

Between February 2008 and February 2010, 49,706 patients were admitted to the emergency department; 542 patients had pneumonia and 75 patients met HCAP criteria. We excluded 21 patients: 13 for exclusion criteria, 5 for technical problems (unavailable fiberscope or operator) and 3 for missing data. Finally, 54 patients with HCAP were included in the study (Figure 2). Patient characteristics are shown in Table 1. A total of 27 patients were hospitalized in the ICU (50%), 9 in the intermediate care department (17%), 11 in the respiratory department (20%) and 7 in other departments (13%) (Figure 2). The mean Fine score was 134 ± 17.5 without antibiotic therapy versus 156 ± 19.5 with previous antibiotic therapy (*P *<0.05). The mean CURB65 score was 1.6 ± 1.

Among the patients, 33 had received antibiotic therapy and 21 did not receive antibiotic therapy before the pathogen identification procedures.

### Diagnostic procedure

All 54 patients had blood cultures and fiberoptic bronchoscopy-guided mini-BAL. We completed 37 mini-BAL procedures in patients with NIV support and 17 in intubated patients with mechanical ventilation support. No complications possibly related to bronchoscopy were reported.

### Microbiological identification rate

**Figure 3:**
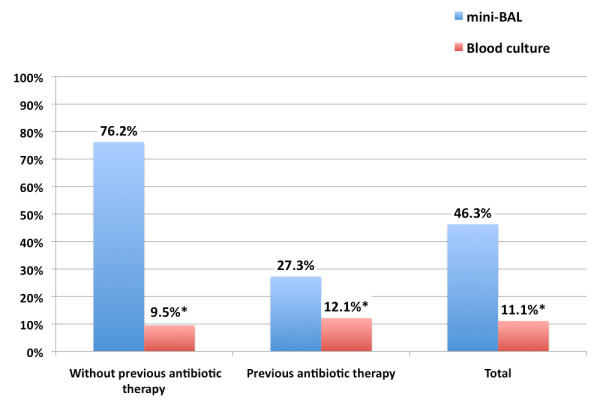
**Bacteriological identification blood cultures versus fiberoptic bronchoscope-guided distal protected small volume bronchoalveolar lavage (FODP mini-BAL)**. *P <0.05

Among the 54 patients, pathogens were identified in 25 patients (46.3%) using mini-BAL, and in 6 patients (11.1%) using blood cultures (*P *< .01) (Figure 3). When both blood cultures and mini-BAL were positive, they always identify the same organism. Bacteriological identification revealed a broad spectrum of bacteria (Table 2). Among 28 identified pathogens, 23 wild-type resistance phenotype or had low levels of drug resistance and 5 were MDR.

**Table 2:** Bacteriological identification with FODP mini-BAL

Pathogens	Number of patients		
	**Without previous antibiotic therapy**	**Previous antibiotic therapy**	**Total**
Pathogens per sample			
1	14	8	22
2	2	1	3
Gram positive pathogens			
Staphylococcus aureus	2	1	3
Streptococcus pneumonia *MDR strain*	5	1	6 *2*
Gram negative pathogens			
Pseudomonas aeruginosa *MDR strain*	4	0	4 1
Haemophilus influenza	1	2	3
Klebsiella pneumoniae	2	1	3
Escherichia coli *MDR strain*	1	2	3 2
Enterobacter aerogenes	1	0	1
Enterobacter cloacae	1	0	1
Serratia marcescens	0	1	1
Proteus vulgaris	0	1	1
Other pathogens			
Legionella pneumophila	0	1	1
Mycobacterium tuberculosis	1	0	1

FODP mini-BAL; fiberoptic bronchoscope-guided distal protected small volume bronchoalveolar lavage

Twenty-one patients had not received previous antibiotic therapy. Among these patients, we obtained bacteriological diagnoses for 16 patients (76.2%) using FODP mini-BAL and for 2 patients (9.5%) using blood cultures (Figure 3). Thirty-three patients had received previous antibiotic therapy before the microbiological diagnostic procedure. We obtained identification for 9 patients (27.3%) using mini-BAL and for 4 patients (12.1%) using blood cultures (Figure 3). The relative risk of non-identification with previous antibiotic therapy versus without previous antibiotic therapy with FODP mini-BAL is 3.3 (95% confidence interval, 1.5 to 7.25).

In the patients with bacteriological identification, we studied the effectiveness of the initial antibiotic therapy. With the broad-spectrum treatment of the study, three initial treatments were ineffective on the pathogen (*M. tuberculosis*, *Escherichia coli *BLSE, and *L. pneumophila*).

In theory, if we used antibiotic treatment of severe community acquired pneumonia, with combined Ceftriaxon/Levofloxacin, six initial treatments were ineffective (three *P. aeruginosa*, *M. tuberculosis*, *E. coli *BLSE, and *L. pneumophila*) and treatments were not optimum for three patients infected by *Staphylococcus aureus*.

### De-escalation rate

When bacteriological identification was obtained, initial antibiotic therapy could be adapted to the antibiogram in 100% of cases.

## Discussion

To our knowledge, this is the first study to focus on bacteriological diagnosis using FODP mini-BAL in HCAP patients admitted to an emergency department. The main result is that this technique is a more effective method to obtain pathogen identification than blood culture (46.3% versus 11.1% *P *< .01). This result is concordant with previous studies. Deschamps *et al*. observed a similar identification rate in the context of hospital-acquired pneumonia with BAL and blood cultures [15]. Pathogen identification is more successful when FODP mini-BAL is performed before antibiotic therapy (76.2% versus 27.3%; *P *< .001; relative risk of non-identification was 3.3 (95% confidence interval, 1.5 to 7.25). This result suggests that if decided, this exam should be completed promptly and before the beginning of antibiotic treatment.

We identified 28 bacteria, 5 of which were MDR. A secondary goal of this study was to evaluate the pertinence of the HCAP categorization in our hospital. We evaluated the empirical antibiotic therapy comparated to antibiogram. If we use severe community acquired pneumonia antibiotic therapy (cefriaxone combined with levofloxacin), 6/28 (21.5%) treatments will be inefficient (3 *P. aeruginosa*, *M. tuberculosis*, *E. coli *BLSE, and *L. pneumophila*). This result justifies HCAP categorization and the ATS strategy with initially broad-spectrum antibiotic therapy. The Falcone *et al.* study indicates that a broad-spectrum empirical approach may be associated with improved outcome and reduction in the length of hospitalization [16].

However, 23 (82%) of the bacterial strains identified had wild-type resistance phenotype or had low-level resistance and 3 (10%) were resistant to our broad-spectrum antibiotics therapy. This result favors the use of an efficient pathogen identification technique to adapt antibiotic therapy with two goals: avoid the economic and ecological cost of an unnecessary antibiotics prescription and be efficient against bacteria resistant to the initial antibiotic therapy. This is why we propose to use early FODP mini-BAL in the emergency department. However, the worse efficiencies of mini-BAL after antibiotic therapy and the logistics required favor performing mini-BAL only for patients without previous antibiotic therapy.

We have to recognize that our local bacterial ecology is quite different from that described in other areas, as in the study by Kollef *et al.* who reported a higher proportion of *S. aureus *and methicillin-resistant *S. aureus *in a multi-institutional database of US acute-care hospitals [3].

Our study shows that fiberoptic bronchoschope-guided mini-BAL is a feasible procedure in patients with HCAP. We experienced no major complications despite the proportion of patients who were more than 80 years old (26%). Some studies have already reported the feasibility and the safety of this technique in a different context. Azoulay *et al.* used fiberoptic BAL (50 mL serum saline) in patients with hypoxemic acute respiratory failure. As in our study, they concluded that this strategy was safe, with no increased risk of endotracheal intubation [17]. In the literature, this procedure is often described under NIV when patients are not intubated. Maitre *et al.* used continuous positive airway pressure (CPAP) during fiberoptic bronchoscopy in hypoxemic patients to prevent subsequent respiratory failure [18]. Da Conceiçao *et al.* performed BAL in hypoxemic and hypercapnic chronic obstructive pulmonary disease patients using bilevel ventilatory support [19].

The use of bronchoscopy allows us to select the sampling site with chest X-ray. The low volume used for mini-BAL (20 ml) probably has a better tolerance than the larger volume used for BAL (250 ml).

Based on our results, we agree with Brito *et al.* when they suggest that HCAP is a heterogeneous disease and that all patients do not need the same broad-spectrum antibiotic therapy [20]. Our bacteriological results are in agreement with this idea. Moreover, a recent study suggested that the HCAP concept does not correlate well with the presence of infection due to a resistant pathogen [5]. From our point of view, this debate promotes the use of an efficient pathogen identification technique to avoid the use of broad-spectrum antibiotics and to de-escalate initial antibiotics as soon as possible [21]. Besides, some authors propose to redefine the concept of HCAP which may contribute to confusion more than provide a guide to pneumonia management, and potentially leads to overtreatment [22]. Achieving bacteriological identification in a larger population study should define new HCAP criteria and adapt empirical antibiotic therapy to these new categories.

Our study has several limitations. It appears that the main limitation of our strategy is the availability of the fiberoptic bronchoscope and an experienced operator when the patient is admitted to the emergency department. Indeed, the examination should be completed promptly after hospital admittance so that antibiotic therapy can be started as soon as possible. We describe our local bacteriological ecology. It has been shown that pathogens and their drug-sensitivity may be different in other areas [3]. An additional limitation is that our study is observational. We did not compare the effectiveness of our strategy regarding outcomes with an antibiotic strategy based on non-invasive pathogen identification. Large scale, multi-center studies are needed to confirm our strategy regarding outcome, as well as ecological and economic costs.

## Conclusions

Our study demonstrates that early FODP mini-BAL is safe and more efficient than blood cultures to identify pathogens and de-escalate antibiotic therapy in patients presenting with HCAP. We demonstrated that HCAP classification is relevant in our hospital. However, other studies are needed to compare the efficiency of this strategy including mini-BAL with a non-invasive strategy including sputum cultures, blood cultures, and an epidemiologic approach in terms of outcome and the economic impact of early antibiotic de-escalation.

## Key messages

• Early FODP mini-BAL is safe and more efficient than blood cultures to identify pathogens and de-escalate antibiotic therapy in the treatment of HCAP (46.3% versus 11.1%, *P *< .01).

• FODP mini-BAL should preferentially be completed promptly and before the beginning of treatment (relative risk of non-identification was 3.3 (95% confidence interval, 1.5 to 7.25).

## Abbreviations

ATS: American Thoracic Society; BAL: bronchoalveolar lavage; HCAP: health care-associated pneumonia; MDR: multidrug-resistant; Mini-BAL: fiberoptic bronchoscope-guided distal-protected small volume bronchoalveolar lavage; NIV: non-invasive ventilation.

## Competing interests

The authors declare that they have no competing interests.

## Authors' contributions

GL: study concept and design, acquisition of data, drafting of the manuscript. BP: study concept and design, acquisition of data, drafting of the manuscript. JB: acquisition of data, drafting of the manuscript. NCA: analysis and interpretation of data. YA: acquisition of data, analysis and interpretation of data. TG: acquisition of data, bacteriological analysis. SP: acquisition of data, bacteriological analysis. EA: acquisition of data, drafting of the manuscript. DK: acquisition of data, study concept and design. EM: Study concept and design, acquisition of data, drafting of the manuscript. PG: study concept and design, acquisition of data. All authors read and approved the final manuscript.
